# The Development and Performance of a Machine-Learning Based Mobile Platform for Visually Determining the Etiology of 5 Penile Diseases

**DOI:** 10.1016/j.mcpdig.2024.04.006

**Published:** 2024-05-01

**Authors:** Lao-Tzu Allan-Blitz, Sithira Ambepitiya, Raghavendra Tirupathi, Jeffrey D. Klausner

**Affiliations:** a Division of Global Health Equity, Department of Medicine, Brigham and Women’s Hospital, Boston, MA; b HeHealth, San Francisco, CA; c Keystone Infectious Diseases, Keystone Health, Chambersburg, PA; d Department of Population and Public Health Sciences, Keck School of Medicine, University of Southern California, Los Angeles

## Abstract

**Objective:**

To develop a machine-learning visual classification algorithm for penile diseases in order to address disparities in access to sexual health services.

**Patients and Methods:**

We developed an image data set using original and augmented images for 5 penile diseases: herpes lesions, syphilitic chancres, balanitis, penile cancer, and genital warts. We used a U-Net architecture model for semantic pixel segmentation into background or subject image, an Inception-ResNet version 2 neural architecture to classify each pixel as diseased or nondiseased, and a salience map using GradCAM++. We trained the model on a random 91% sample of the images and evaluated the model on the remaining 9%, assessing recall (or sensitivity), precision, specificity, and F1-score. As of July 1st 2022, the model has been in use via a mobile application platform; we assessed application usage between July and October 1, 2023.

**Results:**

Of 239 images in the validation data set, 45 (18.8%) were of genital warts, 43 (18%) were of herpes simplex virus infection (ranging from early vesicles to ulcers), 29 (12.1%) were of penile cancer, 40 (16.7%) were of balanitis, 37 (15.5%) were of syphilitic chancres, and 45 (18.8%) were nondiseased images. The overall accuracy of the model for correctly classifying images was 0.944. There were 2640 unique submissions to the mobile platform; among a random sample (n=437), 271 (62%) were from the United States, 64 (14.6%) from Singapore, 41 (9.4%) from Canada, 40 (9.2%) from the United Kingdom, and 21 (4.8%) from Vietnam.

**Conclusion:**

We report on the development of a machine-learning model for classifying 5 penile diseases, which exhibited excellent performance.

Sexually transmitted infections cause major disruptions to sexual, reproductive, and neonatal health, with a range of consequences among those with untreated infection including infertility,^1^ an increased risk for HIV transmission and acquisition,^2^^,^^3^ cervical cancer,^4^^,^^5^ neonatal blindness,^6^ and fetal demise.^7^ Further, such infections are highly prevalent. The rates of syphilis, caused by *Treponema pallidum*, have risen dramatically over the past several years.^8^ The cumulative lifetime incidence of human papilloma virus infection is estimated to be as high as 10% globally.^9^ Herpes simplex virus 2, the leading cause of genital herpes, is estimated to affect 12% of individuals aged between 15 and 49 years in the United States^10^ and 13% globally.^11^

Resource-limited settings often experience disproportionate rates of sexually transmitted infections.^12^ Such areas, however, often lack laboratory infrastructure to support pathogen-specific detection and treatment. For many sexually transmitted diseases, characteristic cutaneous manifestations may be sufficient to support diagnosis independent of microbiologic testing—such as herpes lesions caused by herpes simplex virus, genital warts caused by human papilloma virus, chancres caused by *T pallidium*, and balanitis, most commonly caused by *Candida* subspecies. However, visual diagnosis requires access to care services and experienced providers, which may be infeasible for patients living in rural areas or places with few trained clinicians. Further, the stigma around sexually transmitted infections can limit care seeking,^13^^,^^14^ as well as appropriate diagnosis and treatment by inexperienced clinicians even once care has been sought.^15^

Innovative technologic solutions can overcome those barriers, permitting increased access to visual disease classification. Machine-learning, a form of artificial intelligence, has been used in an array of medical contexts to augment clinical decision making, including for sexual health care.^16^ Machine-learning has been used to predict who might benefit most from pre-exposure prophylaxis to HIV,^17^ estimate the county-level rates of syphilis based on social media behaviors,^18^ and predict an individual’s risk for sexually transmitted infections based on responses to routine surveys.^19^ During the 2022 mpox outbreak, machine-learning models were developed to visually classify mpox; those models reported a range of accuracy between 78% and 98%.^20^ Leveraging a similar approach, we aimed to develop a machine-learning platform to classify 5 penile disease states: herpes lesions, syphilitic chancres, balanitis, penile cancer, and genital warts.

## Patients and Methods

In this study, we describe the development of a machine-learning algorithm, which includes clinical image data set generation; the development of models for semantic segmentation, classification, and salience modules; and finally training the algorithm and assessing performance on a subset of the image database.

### Incorporation Into a Mobile Platform: HeHealth

We developed a free mobile application interface to house the machine-learning model. Users could download the application, respond to a 4-question survey, and submit anonymous images. Survey questions included country of residence, year of birth, presence of any symptoms (penile pain, penile discharge, and pain/burning on urination), and last sexual contact. The platform accepted images submitted with any smartphone and provided users results of the disease classification within seconds. For users who submitted images classified with any of the 5 diseases, the platform provided educational material specific to the disease (eg, common symptoms, types of confirmatory testing needed, and treatment options) and provided additional resources to facilitate linkage to care.

A free, basic version of the mobile platform using an image classification model was launched in July 2022. That pilot launch enabled us to source clinical images from the mobile platform for subsequent iterative model development described further. The updated model was launched in July 2023. We used a random sample of submissions between July and October 2023 to evaluate the distribution of current users. We present user responses to survey questions during that period, stratified by country of residence.

### Clinical Image Data Set Generation

We developed a repository of clinical images over 2 phases. Phase 1 consisted of obtaining clinical images from physicians of 5 penile diseases: herpes lesions, syphilitic chancres, balanitis, penile cancer, and genital warts. Clinicians who contributed images were specialists in infectious diseases, sexually transmitted diseases, dermatology, or family practice with a special interest in sexually transmitted diseases. Those clinicians practiced in 6 different countries: India (n=4), Sri Lanka (n=3), Singapore (n=3), Australia (n=3), the United States (n=1), and the United Kingdom (n=1). In addition, we used a custom-built web-scraping tool to download images freely available on the internet. In total, we curated over 20,000 images.

Images were initially screened for the presence of any disease. If disease was present, 1 expert clinician reviewed and classified the image into 1 of the 5 penile diseases of interest or excluded the image if it depicted a nonrelevant disease or was nonspecific. All images classified as 1 of the 5 penile diseases of interest were then evaluated by a second expert clinician. If the second reviewer agreed with the first determination, the image was then labeled with distinguishable disease characteristics and included in the training image data set. If the second reviewer disagreed with the disease classification, the image was considered ambiguous and thus excluded. The expert clinicians who contributed to labeling and classifying the clinical images were the same clinicians who provided clinical images. Phase 1 resulted in 1000 labeled and categorized images (630 contributed from physicians and 370 sourced from the internet).

Phase 2 involved 3 components. First, we sought additional deidentified clinical images from clinicians in Singapore, India, and Sri Lanka. Second, we publicly sourced images from the mobile application interface, which was predominantly used in North America. All images sourced from the mobile application were evaluated by at least 2 expert clinicians and included only if both clinicians agreed on the diagnosis. Finally, we implemented layered image augmentation to offset the unequal distribution of clinical images across the 5 disease categories. Layered image augmentation involved 2 stages: first, we conducted manual image augmentation by extracting specific visually recognizable disease patterns from the existing clinical image data set, accounting for disease location, skin complexation, and penis orientation. Those disease patterns were then overlay on top of a separate data set of nondiseased penises to create additional, artificially modified images of the 5 penile diseases. For each manually augmented image, an expert clinician verified the validity of the image. Subsequently, we implemented automated random image augmentation, which randomly applied selected transformations to each image during the model training process to generate permutations of a given image. Those permutations included rotations, rescaling of the image size, shifts in the x and y axes, changes in brightness, vertical or horizontal flips, and alterations in size, and color. We used Generative Adversarial Networks technology to generate the modified images.^21^ Supplemental Figure 1 (available online at https://www.mcpdigitalhealth.org/) shows an example of permutated images using various transformations included in the augmented image data set for model training.

**Figure 1:**
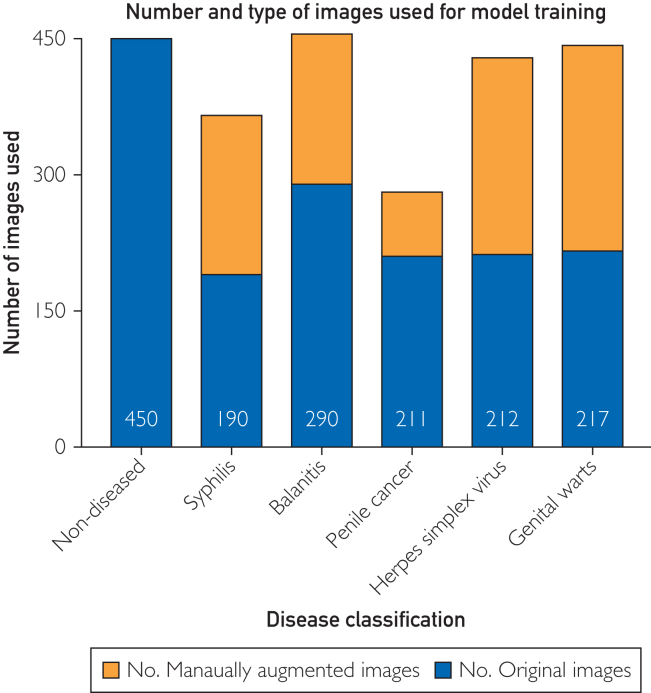
The number of original and manually augmented images used across the 5 penile pathology classifications of interest (syphilitic chancres, balanitis, penile cancer, herpes lesions, and genital warts). Numbers in blue bars reflect number of original images.

Phase 2 resulted in 2627 images, of which 1570 (59.8%) were original submissions (50% contributed by clinicians, 50% sourced through the mobile application) and 1057 (40.2%) developed via image augmented (Figure 1).

### Model Development: Semantic Segmentation, Classification, and Salience Modules

We used semantic image segmentation, in which each pixel of a given image was categorized into 1 of the several predefined classes to isolate the visually distinguishable diseased regions for each of the 5 penile disease classifications and to disregard the background and nondiseased penis components. First, we classified pixels as either background or subject image using a U-Net architecture model.^22^ To further classify images, we applied the Inception-ResNet version 2 neural architecture,^23^ a pretrained model developed to classify images from a generic data set and learn visual correlations. We used a common image optimizer, the Adam optimizer,^24^ to reduce information losses via assigning weights to the images classified by the Inception-ResNet version 2 model. That optimizer permitted further focus to improve identification of images of a penis. The model was trained to conduct 2 predictions: the penile disease classification and salience of the classification prediction (or quantified conspicuity of each pixel in the image).^25^ We mapped the predicted salience using GradCAM++, which facilitated visual explanations of model prediction results and localization of identified pathology (Supplemental Figure 2, available online at https://www.mcpdigitalhealth.org/).^26^

We further optimized the model using an image processing module to focus on diseased regions and reduce background signals. That was done by using the segmentation mask determined by the semantic segmentation module within a bounding box. The bounding box was computed to encompass the salient regions identified by the initial model. The pixels within the bounding box were then re-input as the image for which classification would be assessed using GradCAM++.

To refine the model, as well as assess model performance, we developed a training data set by randomly selecting 91% of the overall images (n=2388; both original and augmented images) over 150 epochs (number of times the model works through the entire training data set). The learning rate and epsilon of the optimizer was set to 0.01 and 0.1, respectively. We trained the model with tesla p100 16-gb graphics processing unit provide by Kaggle cloud platform. We then evaluated the performance of the model on the remaining 9% of images (n=239). Evaluation metrics included recall (or sensitivity), precision (or positive predictive value), specificity, and F1-score,^27^ calculated for each disease classification using the equations given further. Overall accuracy of the model was defined as the F1-score averaged across each disease class.

### Equations

$$Recall\;\left(Sensitivity\right)=\frac{True\;Positive}{\left(True\;Positive+False\;Negative\right)}$$

$$Precision=\frac{True\;Positive}{\left(True\;Positive+False\;Positive\right)}$$

$$F1\;Score=2*\frac{\left(Precision*Recall\right)}{\left(Precision+Recall\right)}$$

### Ethical Approval

The Mass General Brigham institutional review board deemed the analysis of deidentified data did not constitute human patients’ research.

## Results

### Performance of the Machine-Learning Algorithm

Of the 239 images in the validation data set, 45 (18.8%) were of genital warts, 43 (18.0%) were of herpes simplex virus infection, 29 (12.1%) were of penile cancer, 40 (16.7%) were of balanitis, 37 (15.5%) were of syphilitic chancres, and 45 (18.8%) were of nondiseased penises. Table 1 summarizes the classification assignment and performance metrics of the model for each category. The overall accuracy of the model for correctly classifying the diseased image was 0.944.

**Table 1:** Outcomes of the Machine-Learning Model for Visually Classifying 5 Penile Diseases

	No. of images (n=239)	True positive	False positive	True negative	False negative	Recall or sensitivity (95% CI)		Precision or positive predictive value (95% CI)		Specificity (95% CI)		F1 score
Genital warts	45	43	7	187	2	0.956	0.849-0.995	0.860	0.764-0.956	0.964	0.927-0.985	0.909
Herpes lesions	43	40	6	190	3	0.930	0.810-0.985	0.870	0.772-0.967	0.969	0.935-0.989	0.917
Penile cancer	29	23	3	207	6	0.793	0.603-0.920	0.885	0.762-0.999	0.986	0.959-0.997	0.932
Balanitis	40	35	1	198	5	0.875	0.732-0.958	0.972	0.919-0.999	0.995	0.972-0.999	0.983
Syphilis	37	32	3	199	5	0.865	0.712-0.955	0.914	0.822-0.999	0.985	0.957-0.999	0.948
Nondiseased	45	44	2	192	1	0.978	0.882-0.999	0.957	0.852-0.995	0.990	0.963-0.999	0.973

### Current Usage of the HeHealth Platform for Classifying 5 Penile Diseases

**Figure 2:**
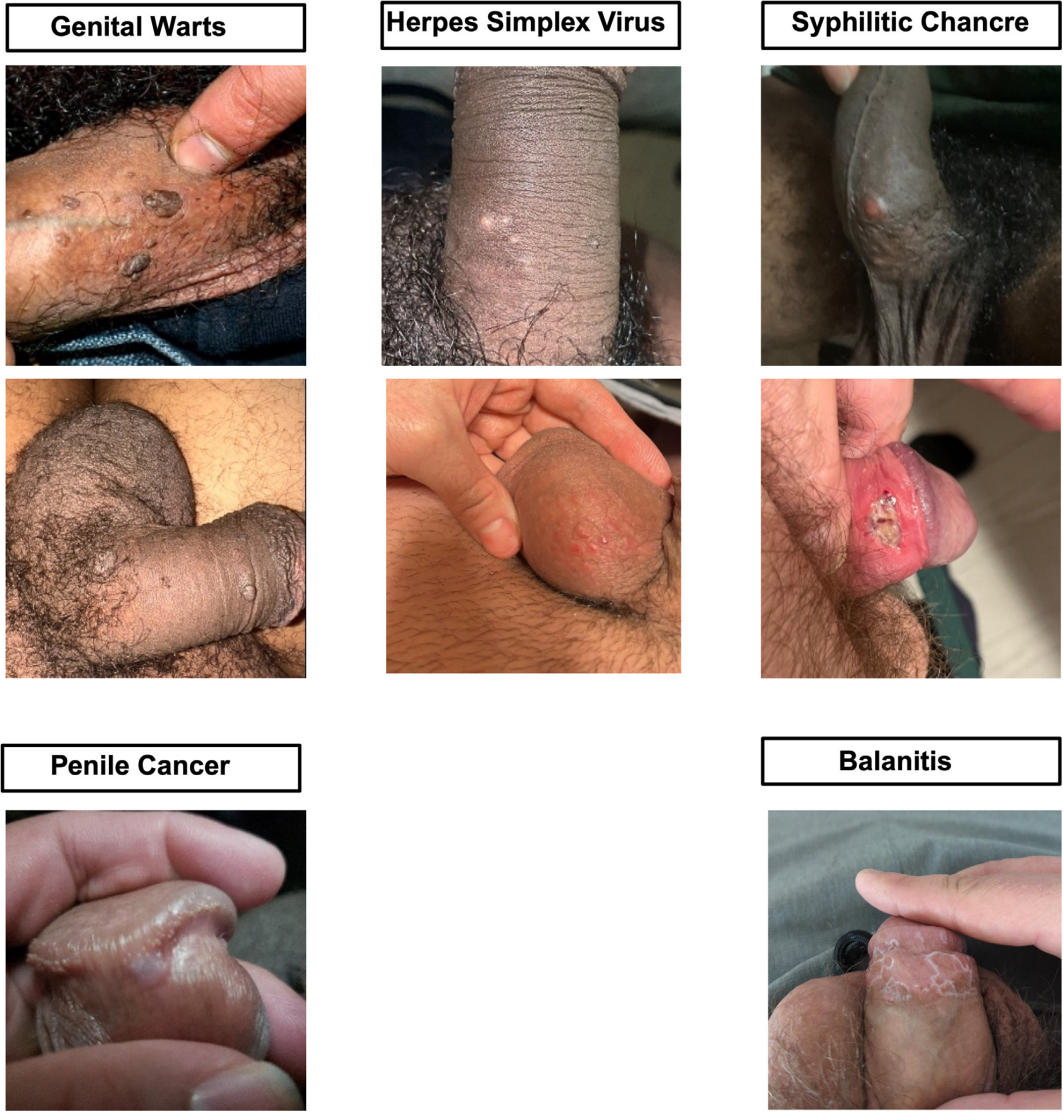
The images submitted by HeHealth users are classified into 1 of the 5 penile disease categories by the model.

As of October 1, 2023, there were a total of 37,100 unique submissions to the HeHealth platform. After the latest software on July 1, 2023 there were 2640 unique submissions. We evaluated a random set of 437 submissions between July and October 2023. Most of those submissions (n=277 [63.4%]) were from individuals between the age of 18 and 30 years. Overall, 271 (62%) came from users in the United States. Table 2 summarizes the distribution of users by country, as well as symptoms reported, and recency of last sexual contact. Figure 2 shows examples of deidentified clinical images submitted by users during that period.

**Table 2:** Distribution and Characteristics of Randomly Selected HeHealth Users Between July and October 2023

	Overall		United States		Singapore		Canada		United Kingdom		Vietnam	
	n	%	n	%	n	%	n	%	n	%	n	%
Total	437	—	271	62.0	64	14.6	41	9.4	40	9.2	21	4.8
Age (y)												
18-30	277	63.4	176	64.9	37	57.8	26	63.4	25	62.5	13	61.9
31-50	144	33.0	87	32.1	23	35.9	14	34.1	13	32.5	7	33.3
>50	16	3.7	8	3.0	4	6.3	1	2.4	2	5.0	1	4.8
Symptoms												
Penile pain	140	32.0	84	31.0	26	40.6	11	26.8	11	27.5	8	38.1
Penile discharge	110	25.2	70	25.8	12	18.8	10	24.4	11	27.5	7	33.3
Pain/burning when urinating	114	26.1	81	29.9	8	12.5	9	22.0	10	25.0	6	28.6
None of the above/other	242	55.4	149	55.0	38	59.4	24	58.5	21	52.5	10	47.6
Last sexual contact												
<1 mo ago	248	56.8	169	62.4	30	46.9	20	48.8	21	52.5	8	38.1
1-3 mo ago	129	29.5	70	25.8	27	42.2	14	34.1	10	25.0	8	38.1
>3 mo ago	49	11.2	27	10.0	4	6.3	6	14.6	8	20.0	4	19.0
Never had sex	11	2.5	5	1.8	3	4.7	1	2.4	1	2.5	1	4.8

## Discussion

We report on the development of a machine-learning model for classifying 5 penile diseases. On the basis of more than 2000 images (original clinical images and augmented images) from a geographically diverse sample, the model demonstrated excellent discrimination between each of the 5 disease classifications and for nondiseased states. Further, the model has been deployed via a free mobile application platform and is currently in use across 5 countries.

Few similar classification models exist for penile diseases. Some models for classifying mpox have found similarly excellent performance with an accuracy greater than 95%.^20^ However, machine-learning has been used across a range of other dermatologic conditions including melanoma, diabetic foot ulcers, psoriasis, and rosacea.^28^ Most of those have used ResNet models, and several paired a ResNet model with a U-Net architecture as done in this study. Those models have reported a wide range of performance (recalls between 69% and 99%; accuracy between 89% and 99%).^28^

Our model exhibited varied performance by disease classification. Model recall (or sensitivity) was high for genital warts and herpes lesions, followed by balanitis and syphilitic chancres, and highest for nondiseased images. Recall was lowest for penile cancer, which may be due to several factors. First, penile cancer may have a more heterogeneous appearance compared with the other disease states. Second, there were fewer augmented images generated for penile cancer generated, which was felt to be specific to the nature of how penile cancer visually presents, with a high probability of distortion within generated images. The lower number of augmented images likely resulted in both a lower overall sample on which to train the model and lower diversity of images.

Model precision (or positive predictive value) was similarly highest for images of a nondiseased penis, although specificity was more than 90% for all 5 diseases. Importantly, this model was trained on images of these 5 disease classifications and nondiseased images. Evaluation of the performance of the model among images of other penile diseases, such as other causes of genital ulcer disease (eg, *Haemophilus ducry* infection), will be essential to validate model specificity. Model training using additional images from a more diverse sample is currently ongoing, which may further improve recall.

Barriers to accessing care for sexual health, including sexually transmitted diseases, are numerous. In both high-resource and low-resource settings, fear of positive test results as well as social stigmatization and shame limit health care seeking.^29^, ^30^, ^31^ In low-resource settings, individuals may not be able to reach a health care center.^31^ Even among those who do, diagnostic options for such diseases are often limited or nonexistent, and provider-level stigma may further limit appropriate treatment.^15^ Therefore, user-guided, mobile, image-based classification tools can facilitate rapid disease classification in various contexts. The mobile application interface is currently in use across 5 countries of varying socioeconomic profiles. Although we did not capture data on access to sexual health services among users of the HeHealth platform, previous studies have shown that such services and expertise are limited Singapore and Vietnam.^32^, ^33^, ^34^ Thus, the uptake of the HeHealth platform in such areas supports the potential deployment of similar tools to augment case identification, facilitate earlier treatment, and reduce both the spread of disease and its consequent morbidity.

Such a user-accessible digital tool is predicated on the near-universal availability of smartphones, which, even in many low-resource areas, are ubiquitous; in 2022 there were an estimated 6.4 billion smartphone mobile network subscriptions worldwide.^35^ However, some settings without smartphones or internet access will not be able to use this tool. Further, correctly classifying the disease must be paired with patient education interventions and linkage to care to facilitate treatment. The HeHealth mobile platform attempts to accomplish both by sending users who are classified as having 1 of the 5 diseases educational material specific to the diagnosis. That additional information includes common symptoms, what type of confirmatory testing would be required, and treatment options. The application also provides links for additional resources to facilitate linkage to care. No outcome data, however, were available among HeHealth users.

Further assessment of the model also remains to be done. The performance of the model thus far has been based on images with a pre-existing classification. Comparisons with gold-standard diagnostics are warranted, such as a comparison of the diagnostic accuracy of the model with that of visual inspection by expert clinicians paired with microbiologic assessment where indicated (eg, herpes simplex virus polymerase chain reaction testing of herpes lesions, fungal scraping for candidiasis, and serology as well as darkfield microscopy for *T pallidum* infection). In addition, follow-up data, if available, could facilitate assessment of the clinical impact of the HeHealth platform. Further model building could seek to include additional disease classifications, such as lichen sclerosis or lichen planus, and/or seek to develop similar models for classifying vulvovaginal diseases.

### Limitations

Our study had several limitations. First, the model was based on images with preassigned classifications by expert clinicians, not by images of diseases confirmed via microbiologic or histologic testing. Similarly, there were no clinical or epidemiologic data available for those images, thus evaluation of model performance by clinical factors (eg, age, country of origin, duration of symptoms, and types of sexual exposures) was not possible. Additionally, the performance of the model was assessed on a relatively small number of images, limiting the precision of our findings. Further evaluation on larger prospective data sets is warranted. Evaluation of model performance across different skin tones and across images of varying quality will be important to assess generalizability and should be the subject of future work. However, given the potential applications of this tool, we feel those limitations do not negate the importance of our findings.

## Conclusion

We report on the development of a machine-learning model for classifying 5 penile diseases. The model was developed using both clinical and augmented images and exhibited excellent performance on a validation data set. That model is currently in use globally, and further assessment of model performance is warranted on larger, more diverse data sets.

## Potential Competing Interests

Dr Allan-Blitz and Dr Klausner are advisors to and have received consulting fees from HeHealth. Dr Ambepitiya is a Medical Executive at HeHealth. The other authors report no conflicts of interest.
