# Supplementary material for: The Development and Performance of a Machine-Learning Based Mobile Platform for Visually Determining the Etiology of 5 Penile Diseases
Source: Mayo Clin Proc Digit Health. 2024 May 1;2(2):280–8. doi: 10.1016/j.mcpdig.2024.04.006 (PMC11975829; doi:10.1016/j.mcpdig.2024.04.006)
Supplement: Appendix [file mmc1.pdf]

Appendix

Supplemental Figure 1: Selected Transformations used for Automated Image Augmentation

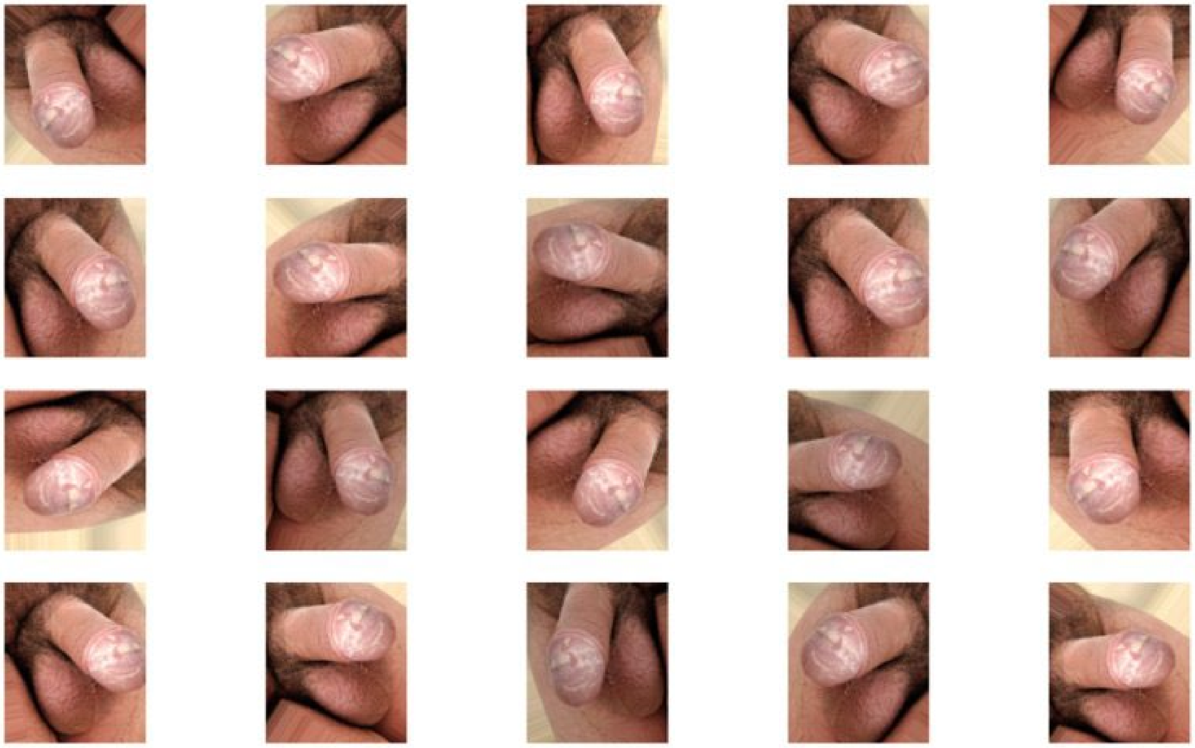

Supplemental Figure 1 Legend: The figure shows various transformations applied to a single image of a diseased penis used for automated image augmentation to offset the unequal distribution of clinical images across the six disease categories

Supplemental Figure 2: Salience Mapping

Panel A **Panel B** **Panel C**

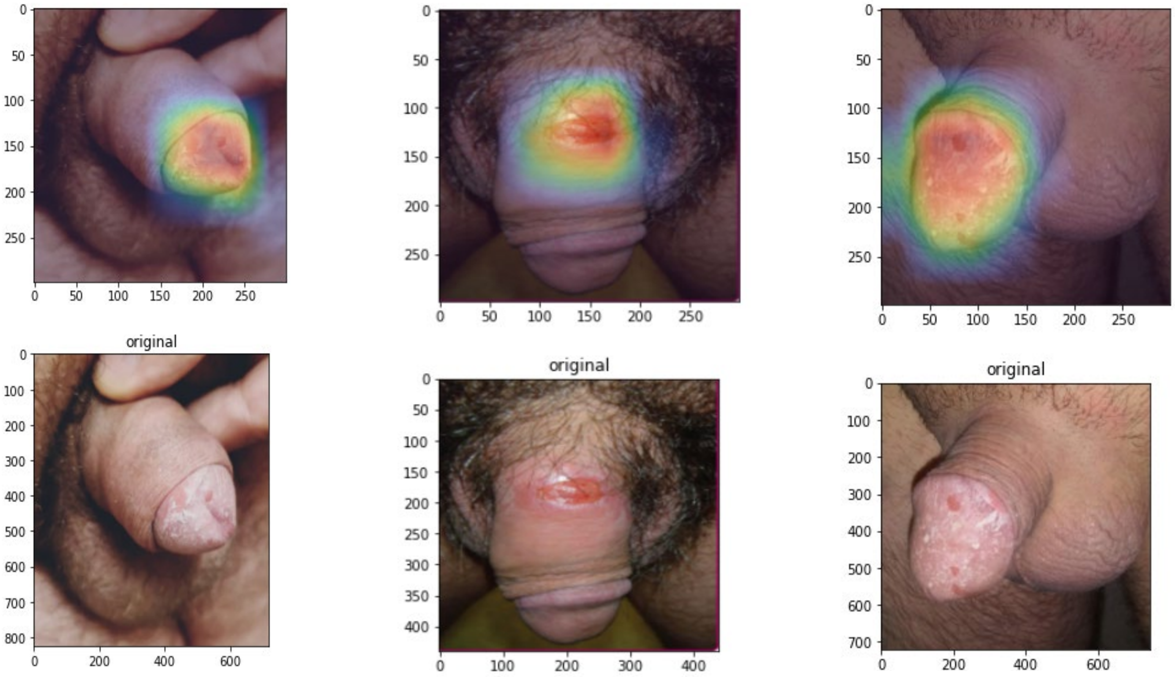

Supplemental Figure 2 Legend: The image shows the salience map for three penile pathologies: balanitis (Panel A), syphilitic chancre (Panel B), and balanitis (Panel C).
